# All-inside suture device is superior to meniscal arrows in meniscal repair: a prospective randomized multicenter clinical trial with 2-year follow-up

**DOI:** 10.1007/s00167-014-3423-5

**Published:** 2014-11-09

**Authors:** Nina Jullum Kise, Jon Olav Drogset, Arne Ekeland, Einar Andreas Sivertsen, Stig Heir

**Affiliations:** 1 Department of Orthopedic Surgery, Martina Hansens Hospital, Postboks 823, 1306 Sandvika, Norway; 2 Department of Orthopedic Surgery, Trondheim University Hospital, Postboks 3250 Sluppen, 7006 Trondheim, Norway

**Keywords:** All-inside, Meniscal repair, Meniscal suture, Meniscus arrow, Biofix^®^, FasT-Fix^®^

## Abstract

**Purpose:**

Multiple techniques and implants are available for all-inside meniscal repair, but the knowledge about their failure rates and functional outcome is still incomplete. The hypothesis was that there might be differences between meniscal arrows and suture devices regarding reoperation rates and functional outcome. Thereby, the aim of this study was to compare clinical results following repair with the Biofix^®^ arrows or the FasT-Fix^®^ suture devices.

**Methods:**

In this RCT, 46 patients were treated either by Biofix^®^ (*n* = 21) or FasT-Fix^®^ (*n* = 25). The main outcome was reoperation within 2 years. Knee function and activity level were evaluated by KOOS and Tegner activity scale.

**Results:**

Twelve out of 46 (26 %) patients were reoperated within 2 years, nine out of 21 (43 %) in the Biofix^®^-group versus three out of 25 (12 %) in the FasT-Fix^®^-group (*p* = 0.018). The relative risk of reoperation was 3.6 times higher for Biofix^®^ compared to FasT-Fix^®^ (95 % confidence interval 1.1–11.5). Both treatment groups had significant increase in all KOOS subscales, but there were no major differences between the groups. The subgroup of reoperated patients differed from the other patients with higher Tegner score preoperatively (median 5 vs. 4) (*p* = 0.037) and at 3-month follow-up (median 4 vs. 3) (*p* = 0.010).

**Conclusions:**

These results indicate that FasT-Fix^®^ suture is superior to Biofix^®^ arrows with significant lower failure rate. Functional outcome did not depend on repair technique. Higher activity score preoperatively and at 3-month follow-up in the reoperated patients indicates that activity level may influence on the risk of reoperation.

**Level of evidence:**

I.

## Introduction

Meniscal repairs are likely to have better long-term outcomes than meniscal resections [17], patients report better functional outcome [11], and it is assumed that repair is superior to resection in preventing osteoarthritis, even if the healing is not complete [19]. Arthroscopic all-inside techniques are often used in preference to outside-in and inside-out techniques because the advantages of shorter operation time and no need for postero-medial or postero-lateral incisions [1].

Studying failure rates in a review of 19 studies, Grant et al. found similar failure rates following inside-out techniques (17 %) compared to all-inside techniques (19 %). The follow-up time of the studies, however, varied from 3 months to 13 years, making the comparison of the techniques hard to interpret [9]. In a meta-analysis, comparing results following open technique, outside-in technique, inside-out technique and different all-inside techniques in 14 cohorts, Nepple et al. [15] concluded with a pooled failure rate of 23 % and no differences between the techniques (failure rates, respectively, 23, 24, 22 and 24 %).

A range of all-inside repair techniques and devices has been introduced. Most clinical studies on all-inside meniscal repair are retrospective, have enrolled small numbers of patients, or have included a range of different all-inside devices [9], and several studies have short observational time only [1, 2, 9]. The first-generation device Biofix^®^ meniscal arrow have been reported with good results with 91 % healing within 4 months [2] and success rates above 90 % at 2–3-year follow-up [8, 12, 18]. Similarly, the second-generation all-inside devices FasT-Fix^®^ are reported with good functional results at 2 years [10] and up to 90 % success of healing at 18 months [13]. In laboratory studies, however, the Biofix^®^ meniscal arrow is shown to have lower pullout strength than the FasT-Fix^®^ suture [4, 27], which have biomechanical properties comparable to inside-out vertical mattress sutures [23].

Thus, the background knowledge for what all-inside technique to choose has been limited and inconclusive. Still, the use of suture devices has increased at the expense of meniscal arrows, based on the assumption that the suture device would provide higher healing rates. In our opinion, there was a need for randomized controlled trials (RCTs) comparing different all-inside techniques. The aim of this RCT therefore was to compare the survival rates and the functional results within 2 years following all-inside meniscal repair using either the Biofix^®^ meniscal arrow or the FasT-Fix^®^ suture. Our working hypothesis was that there might be differences between biodegradable meniscal arrows and suture devices regarding reoperation rates and functional outcome.

## Materials and methods

During 2006–2010, 46 patients enrolled to Martina Hansens Hospital (39 patients) and Trondheim University Hospital (seven patients) with vertical longitudinal meniscal tears eligible for arthroscopic all-inside meniscal repair were included in this prospective randomized double-blinded study. The patients were block randomized (blocks of ten) to arthroscopic meniscal repair with either Biofix^®^ or FasT-Fix^®^ all-inside devices using the “envelope method”, and they were blinded for the treatment choice. The post-operative rehabilitation program was identical in the two treatment groups.

**Fig. 1:**
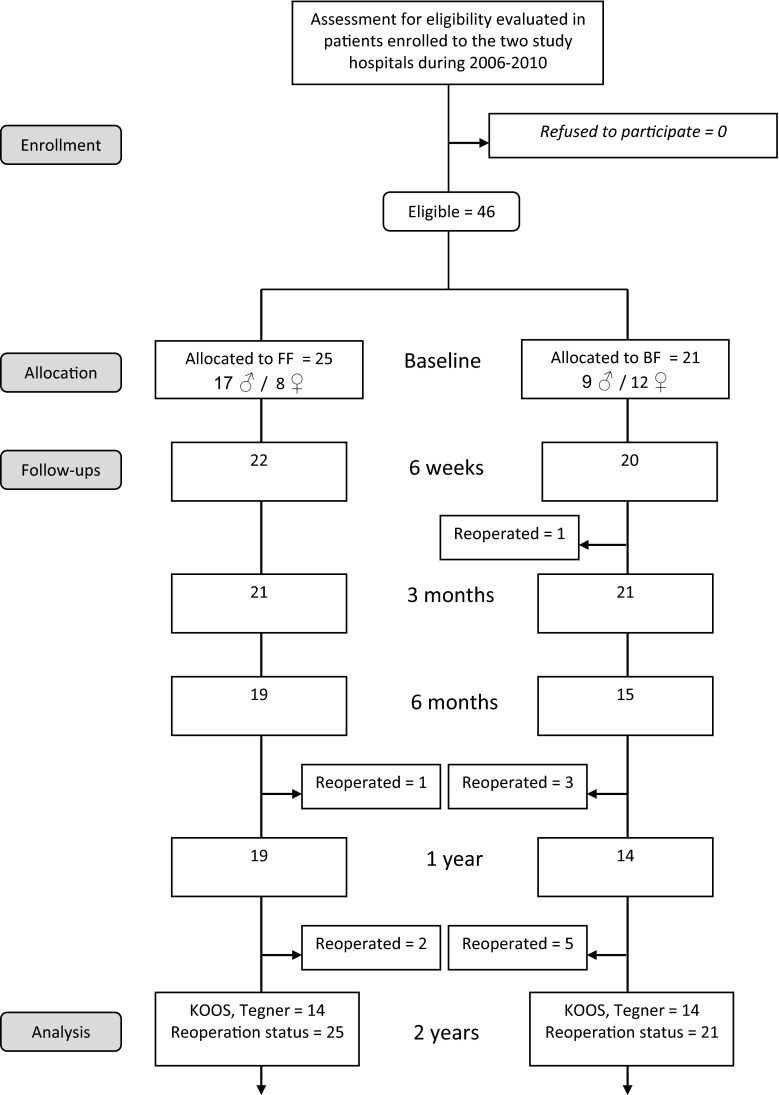
Flow chart

Blinded observers performed post-operative follow-ups after 6 weeks, 3 and 6 months, and 1 and 2 years. Symptoms (e.g. pain, stiffness, locking) and clinical findings (e.g. range of motion, swelling) and potential complications were recorded. All patients that dropped out at 2-year follow-up were interviewed by telephone to verify the reoperation status. The flowchart of patients is shown in Fig. 1.

The main endpoint of the study was reoperation within 2 years as a consequence of complaints due to rerupture or impaired primary healing. Reoperations were recorded when patients had recurrent symptoms of meniscal lesions (e.g. pain, clicking, locking) with clinical indication for reoperation, and the reoperation led to total or partial resection of the incident meniscus tear.

Secondary endpoints were knee function and activity level measured by Knee Injury and Osteoarthritis Outcome Score (KOOS) [21] and Tegner activity scale [24]. KOOS is validated for patients with meniscal tears and osteoarthritis and consists of 42 questions in five categories: pain, other symptoms (Symptoms), activities of daily life (ADL), sport and recreation (Sport) and quality of life (QOL) [21, 22]. Scores in each subscale are transformed to a 0–100 scale, where zero represents extreme knee problems and 100 represent no knee problems [21]. A difference of 8–10 points is regarded as a clinical relevant difference [22]. The Tegner activity scale is graded from 1 through 10, according to the patients self-esteemed level of activity. Ten points are referring to pivoting sports activities (soccer) at international level, five points refer to heavy activities like chopping wood and one point refers to easy house cleaning.

This study was approved by the Regional Ethical Committee for South Eastern Norway (Registration Number 1.2005.2304) and has been performed in accordance with the ethical standards laid down in the 1964 Declaration of Helsinki. All patients gave their informed consent prior to their inclusion in the study.

### Subjects and interventions

Inclusion criteria were patients aged 18–40 years with an MRI-verified vertical, longitudinal meniscal tear, 10–40 mm long, located in the peripheral or the middle third of the meniscus, with a preserved central bucket handle eligible for reduction and repair with all-inside technique. The patients had no conflicting comorbidity, drug addiction or psychiatric conditions affecting surgery or post-operative rehabilitation regime. Exclusion criteria were focal cartilage lesions or osteoarthritis grade 3–4 according to the revised International Cartilage Repair System (ICRS) classification system [5] in an area larger than 1 cm^2^ in the actual knee, ligament tears except tear of the anterior cruciate ligament (ACL) and/or grade 1 tear of the medial collateral ligament (MCL).

The surgery was performed according to standard procedures for knee arthroscopy. When the indications for repair were confirmed, the randomization envelopes were opened. The meniscal tears were debrided with diamond rasp, and by use of adjusted instruments for each device, the implants were inserted on both surfaces of the menisci, seeking adequate adaption and compression of the tear surfaces.

Post-operatively, the patients were instructed to use crutches with partial weight bearing (within 20 kg load) the first 6 weeks post-operatively with unlimited joint range of motion. Flexion up to 90° with concomitant weight bearing was allowed from 6 to 12 weeks post-operatively. After 12 weeks, the patients were allowed to return to sport activities. Patients with ACL tears had concomitant ACL reconstruction or were stabilized in a brace until ACL reconstruction was performed.

### Statistical analysis

The frequency of reoperation after meniscal repair with all-inside devices is reported ranging from 57 to 91 % [2, 13, 26]. In this study, a difference in reoperation rate greater than 10 % was considered as a clinical important difference. With a power of 0.90, a level of significance of 0.05 and standard deviation (SD) of 15, 100 subjects (50 in each group) were needed to detect a 10 % difference. With a 20 % suggested dropout rate at 2 years, the plan was to include 120 patients (60 in each group). However, we chose to interpret our own data at the inclusion of 46 patients, since new information from other studies revealed favourable results using suture techniques compared to meniscal arrows [7, 10]. Based on the results of our own preliminary data, we found it unethical to continue the recruitment of patients. Thus, the total number of patients is 46.

Statistical analyses were performed with IBM SPSS Statistics^®^ (v.21). The value *p* < 0.05 was considered statistically significant and *p* < 0.01 was considered highly significant. Comparisons between groups were performed using the Chi-square test for categorical data and using the Student *t* test or the Mann–Whitney *U* test for continuous data, depending on whether normality could be assumed. Due to low sample sizes, KOOS and Tegner scores are presented with median and range values, although tests showed normality. Logistic regression analysis was performed with reoperation status as the dependent variable and gender, method for meniscal repair and comorbidity in the same knee as independent variables. Time to reoperation was estimated by the Kaplan–Meier method, and differences between the treatment groups were compared using the Log-Rank test. Both intention-to-treat and per-protocol analyses were performed.

## Results

**Fig. 2:**
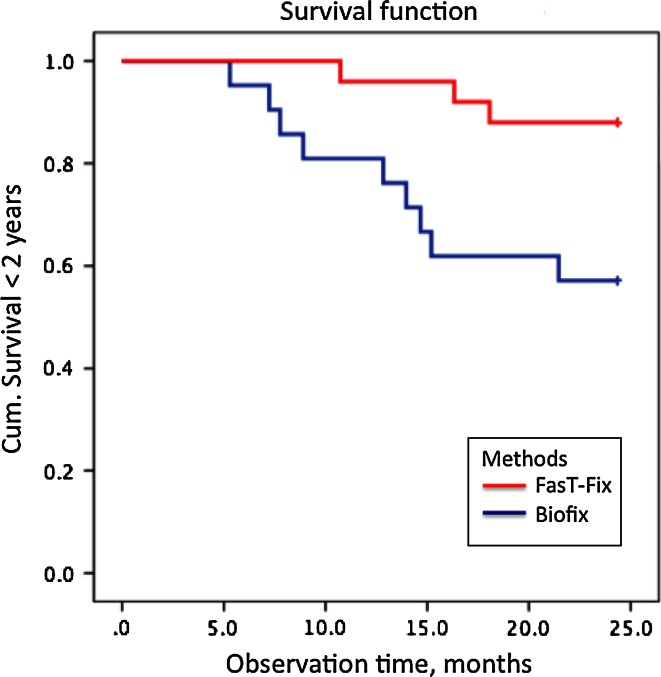
Survival function following meniscal repair with Biofix^®^ meniscal arrows and FasT-Fix^®^ sutures. Each step represents a reoperation due to rerupture or failed primary healing

The baseline data for the two intervention groups were similar (Table 1). Forty-six patients (26 men, 20 women) with median age 25.7 years (range 18.7–40.0) were reviewed 2 years after meniscal repair with either Biofix^®^ arrows or FasT-Fix^®^ sutures. Twelve out of 46 patients (26 %) underwent reoperation within 2 years; nine out of 21 (43 %) patients in the Biofix^®^-group and three out of 25 (12 %) patients in the FasT-Fix^®^-group (*p* = 0.018). The relative risk of reoperation was 3.6 times higher for patients in the Biofix^®^-group compared to the FasT-Fix^®^-group (95 % confidence interval 1.1–11.5). The survival curves for the two repair techniques are shown in Fig. 2.

**Table 1:** Baseline data

Baseline	Biofix^®^-group	FasT-Fix^®^-group
Number	21 Patients	25 Patients
Age		
Median (range)	26.9 years (19.4–39.8)	25.5 years (18.7–40.0)
Gender	9 Men/12 women	17 Men/8 women
Knee	9 Right/12 left	16 Right/9 left
Meniscus	20 Medial/1 lateral	24 Medial/1 lateral
Length of meniscal tear		
Median (range)	20–30 mm (10–40 mm)	20–30 mm (10–40 mm)
ACL pathology Total *N*	5 Patients	7 Patients
Earlier ACL-reconstructed *N*	2 Patients	3 Patients
Concomitant ACL-reconstructions *N*	2 Patients	3 Patients
Remained untreated *N*	1 Patient	1 Patient
Other concomitant injuries	Cartilage injuries 2 patients Osteoarthritis 2 patients	Cartilage injuries 4 patients
Time injury-surgery		
Median (range)	4.5 months (0.2–29.7)	4.1 months (0.2–120.0)

The median time from operation to reoperation was 1.1 years (range 0.4–1.8) in the Biofix^®^-group and 1.3 years (range 0.9–1.5) in the FasT-Fix^®^-group. Eight of the 12 reoperated patients (67 %) reported gradual recurrence of the meniscal symptoms, 3 reported minor traumas and one reported a high energetic valgus trauma. Analysing comorbidity, age, gender and meniscal repair techniques in a logistic regression model with reoperation status as the dependent variable revealed that the meniscal repair technique was the only variable influencing the end point (reoperation) (*p* = 0.019).

**Fig. 3:**
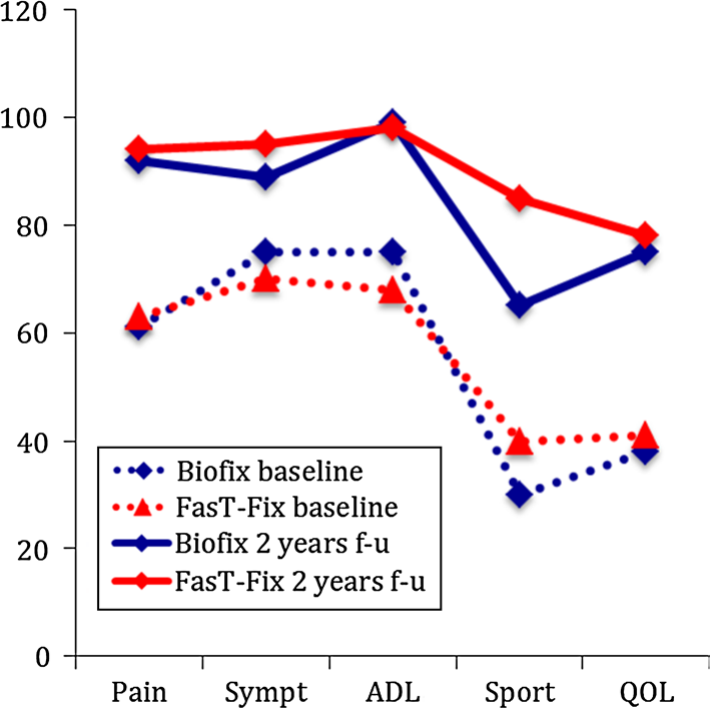
KOOS profiles (expressed as median values) for the two treatment groups preoperatively and at 2-year follow-up (*p* < 0.01 for each subscale in both groups)

**Fig. 4:**
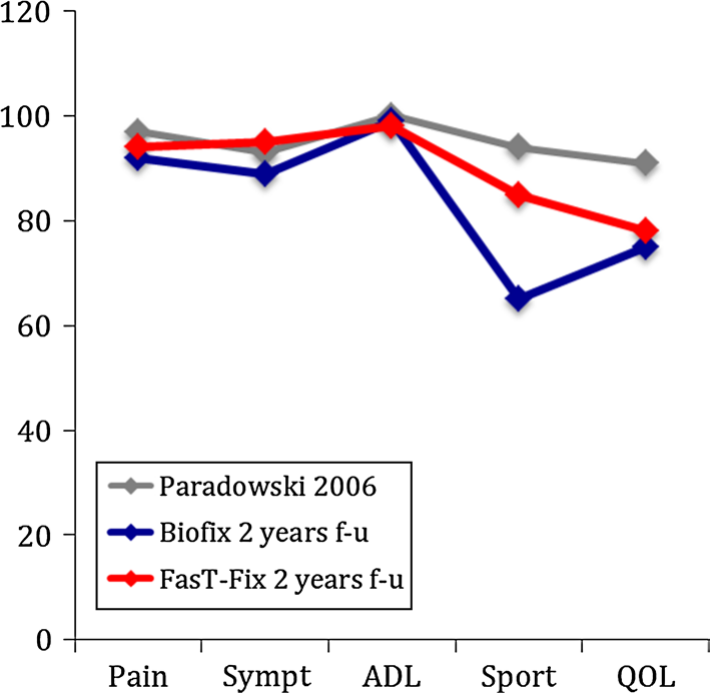
The KOOS profiles (expressed as median values) of the treatment groups at 2-year follow-up did not differ significantly from the KOOS profile of a reference population (*grey line*)

For both treatment groups, there was clinical relevant and statistical highly significant (*p* < 0.01) increase in all KOOS subscales, from baseline to 2-year follow-up (Fig. 3). Comparing the KOOS profiles in the two treatment groups at 2-year follow-up with the profile from a slightly younger reference population [16] revealed that the patients’ KOOS at 2 years were similar to the KOOS profiles of the reference population [16] (Fig. 4). Between the treatment groups, there were no major differences in any KOOS subscale or Tegner score at any follow-ups (Table 2).

**Table 2:** KOOS and Tegner activity scale scores at baseline and follow-ups at 3 and 6 months and 2 years in the two treatment groups

Scores	Group	Baseline	3-month follow-up	*p*	6-month follow-up	*p*	2-year follow-up	*p*
KOOS Pain Median (range)	Biofix^®^	61 (36–86)	72 (53–97)	n.s	94 (54–100)	0.041	92 (69–100)	n.s
	FasT-Fix^®^	63 (31–94)	83 (72–92)		82 (47–97)		94 (25–100)	
KOOS Symptoms Median (range)	Biofix^®^	75 (46–79)	71 (46–96)	n.s.	86 (54–100)	n.s	89 (68–100)	n.s
	FasT-Fix^®^	70 (28–89)	80 (68–86)		77 (61–96)		95 (57–100)	
KOOS ADL Median (range)	Biofix^®^	75 (41–99)	94 (56–100)	n.s.	100 (71–100)	n.s	99 (78–100)	n.s
	FasT-Fix^®^	68 (54–100)	91 (78–99)		88 (57–100)		98 (44–100)	
KOOS Sport-Rec Median (range)	Biofix^®^	30 (0–55)	40 (5–85)	n.s.	75 (5–95)	n.s	65 (25–100)	n.s
	FasT-Fix^®^	40 (0–90)	60 (0–75)		68 (35–95)		85 (50–100)	
KOOS QOL Median (range)	Biofix^®^	38 (6–63)	50 (19–75)	n.s.	81 (6–94)	n.s	75 (25–100)	n.s
	FasT-Fix^®^	41 (0–83)	47 (31–88)		63 (31–94)		78 (6–100)	
Tegner activity Score Median (range)	Biofix^®^	4 (0–9)	4 (0–9)	<0.01	4 (2–9)	0.046	5 (2–9)	n.s
	FasT-Fix^®^	4 (0–10)	3 (0–9)		3 (0–9)		4 (1–9)	

Results from follow-ups at 6 weeks and 1 year are not listed because there were no statistical differences between the groups

Analysing the group of patients that was reoperated within 2 years, comparing them to the group of patients that was not reoperated, revealed that the group of reoperated patients had higher Tegner activity score preoperatively (median 5 vs. 4) (range 3–9 vs. 1–9) (*p* = 0.037) and at 3-month follow-up (median 4 vs. 3) (range 2–9 in both groups) (*p* = 0.010). Except for the higher Tegner score, these patients did not differ from the other patients regarding baseline data or the other variables at follow-up.

Among the 46 patients included in this RCT, one patient in the FasT-Fix^®^-group suffered from a purulent arthritis caused by Staphylococcus aureus diagnosed 2 weeks post-operatively. He was treated successfully with surgical lavage where the implants were left in place and with intravenous and oral antibiotics. Another patient was diagnosed with deep vein thrombosis and pulmonary embolus post-operatively and was treated with thrombolytic medication for 6 months. None of these patients underwent further reoperations. There were no other major complications, and there were no complications related to the implants.

## Discussion

The main finding of this prospective randomized study was a 3.6 times higher risk of reoperation within 2 years following meniscal repair with Biofix^®^ meniscal arrows compared to repair with FasT-Fix^®^ meniscal suture. The reoperation rates are consistent with earlier studies [7, 10, 13] and give support to today’s clinical practice where biodegradable arrows are used less frequent.

Logistic regression analysis demonstrated that neither comorbidity in the same knee, nor age nor gender influenced on the risk of reoperation. Only the choice of meniscal repair device was found to have an impact on the outcome. The post-operative regimes and rehabilitation were identical in the two groups, and the surgical techniques were similar except for the procedures directly related to the implant. Hence, the difference in risk of reoperation seems to be dependent on the implant only. Biomechanical laboratory studies have shown that Biofix^®^ meniscal arrows have lower pullout strength and less flexibility over the repair than FasT-Fix^®^ meniscal suture in cadaver knees [4, 27]. In addition, Biofix^®^ is biodegradable and FasT-Fix^®^ is not. It is possible that the combination of lower pullout strength, less flexibility and biodegradation of the implants leads to lower stability and thereby higher risk of failure in the Biofix^®^-group than in the FasT-Fix^®^-group. It is also possible that the FasT-Fix^®^ devices tends to keep the reduced meniscal fragments more anatomically in place over a longer period of time than the Biofix^®^ arrows, which possibly may reduce or delay reonset of symptoms in patients even if the meniscal tear is not healed.

Another finding was that patients 2 years after meniscal repair—using either method—had knee function assessed by KOOS similar to that of a reference population [16]. The study was underpowered to explore possible between-intervention group differences in patients’ function assessed by KOOS. Thus, no statistically significant differences were revealed within 2 years post-operatively, except for the KOOS subscale Pain at 6 months where patients operated with Biofix^®^ scored better than patients operated with FasT-Fix^®^ (*p* = 0.041) (Table 2). The mean difference was, however, only 10 points, which is recognized as being a borderline value for what is considered clinically relevant [22]. This result should therefore be interpreted with care. However, we also found that Biofix^®^-patients had a significant higher Tegner score at 3 and 6 months compared to the FasT-Fix^®^-patients (*p* < 0.01 and *p* = 0.046, respectively) (Table 2). Neither should this be emphasized heavily, but one explanation may be that the FasT-Fix^®^ sutures give rise to irritation in the joint capsule due to traction of the anchors and thereby pain and reduced activity level, whereas the Biofix^®^ arrows do not since they are mainly located inside the menisci.

The post-operative rehabilitation program was equal for all included patients except for the five who went through simultaneous ACL reconstruction and the two who later decided not to go through reconstruction. These seven patients, however, were equally distributed in the two treatment groups. In the literature, there is no consensus concerning rehabilitation regimes following meniscal repair [3], but during the last decades, the trend is towards less restrictive regimes. The patients in this study were instructed to use crutches with partial weight bearing the first 6 weeks post-operatively but had no restrictions in range of motion without weight bearing. A recent study shows, however, that avoiding weight bearing after repair with FasT-Fix^®^ meniscal suture is unnecessary [25], and a laboratory study concludes that even high flexion is safe, but only when performed in closed-chain exercises [14].

The patients in this study were not allowed to return to sports activities until 12 weeks post-operatively. In spite of this, the group of patients that later went through reoperation, had median Tegner activity score 4 at 3 months, compared to the rest of the patients who had median score 3. A Tegner score of 4 corresponds to activities like moderately heavy labour, cycling, cross-country skiing and jogging [24]. These activities are not recommended for the first 12 weeks post-operatively. It is therefore reasonable to assume that some patients were back to knee demanding activities earlier than prescribed and that this may have contributed to failure in the healing of the meniscal repairs. However, these numbers should be interpreted with care as well, since in both groups, the scores showed a wide range (from the value 2–9). Moreover, the subgroup of patients later being reoperated had a higher median Tegner activity score at baseline compared to those who were not reoperated, again however, with wide ranges (3–9 and 1–9, respectively). It might be possible that individuals with higher physical activity level present higher demands for knee function. They may therefore have a lower threshold for re-consulting the surgeon if they have recurrence of meniscal symptoms, which may possibly result in a reoperation. Patients with lower knee demanding activity levels may accept recurrence of meniscal symptoms better and therefore avoid reoperations. Nevertheless, these findings support the idea of more restrictive return to sports activities, and we suggest that these arguments should be emphasized even stronger for more active individuals.

One strength of the current study was the design. The trial was prospective, randomized and double blinded; neither the patients nor the follow-up examiners knew which device had been used. The time to final follow-up was longer than in previous studies [2, 13, 26], and the main end point—reoperation—is absolute and assumed to be more accurate than the diagnosis of rerupture assessed by e.g. MRI, which is shown to have low reliability [6, 20]. For the main endpoint—reoperation within 2 years—there were no missing data.

Knee function and activity level, measured by KOOS and Tegner activity scale, were the secondary outcomes in this study. KOOS is a widely used self-assessment questionnaire. The score is valid and reliable for patients with different knee injuries, including meniscal tears and ACL tears [22]. In this study, the Tegner activity scale was presented as a self-assessment questionnaire as well, for which it is not validated. Of course this excludes researcher bias, but on the contrary, it opens for the possibility that patients may tend to score themselves to improperly higher or lower activity levels, depending upon characteristics like personality, self-image and ambition level.

The study has the following limitations: the number of patients is relatively low, the inclusion period was quite long, and there were several participating surgeons and the distribution of patients from the two hospitals was rather skew. The low number of patients was the result of stopping inclusion by ethical reasons with a considerable lower number of patients participating than actually planned. However, the difference in the failure rate between the two groups turned out much higher than estimated in the power analysis performed prior to starting the study. Thus, the power for revealing a statistically significant and clinically relevant difference in the main outcome between the treatment groups turned out large enough. Whether the secondary outcomes (knee function and activity level) would have shown some statistically significant differences given a larger number of patients, remains unknown.

None of the patients had MRI examinations at 2-year follow-up. MRI might have given some additional information on the status of the meniscal healing of those patients not being reoperated. On the other hand, there are some pitfalls using MRI as a criteria for meniscal healing, since the overall sensitivity and specificity for diagnosing a meniscal tear on conventional MRI is reported to be as low as 79 and 88 % [6]. Following meniscal repair, the MRI-diagnostics is even more challenging, and false positive tears may be reported still after healing of the meniscus [6, 20].

Despite the limitations in this study, it verifies that meniscal repair with an all-inside suture device is superior to meniscal arrows regarding reoperation rate during the first 2 years post-operatively. The 3.6 times higher risk of reoperation following repair with arrows will presumably have important impact on the choice of methods for meniscal repairs in clinical practice. Longer term follow-up studies are necessary to determine the influence of the different methods on degenerative changes over time.

Saving meniscal tissue is important [17], and all-inside meniscal repair has benefits compared to outside-in and inside-out techniques [1]. Therefore, in clinical practice, all-inside techniques are often used and the technique and device seems to be important. The results of this study may primarily contribute to better knowledge in choosing among the wide range of devices available today and secondary in designing new devices in the future. Thus, this study represents a high clinical relevance.

## Conclusions

This study showed a significant lower rate of reoperations following meniscal repair with FasT-Fix^®^ all-inside suture compared to Biofix^®^ all-inside meniscal arrows. Functional outcome was not dependent upon repair technique, but the higher Tegner scores preoperatively and at 3 months post-operatively in the group of patients being reoperated at a later stage, imply that activity level may influence on the risk of reoperation. This study strongly advocates the use of a suture device instead of meniscal arrows in all-inside meniscal repair. It also suggests a restrictive activity level within the first 3 months of rehabilitation.
